# Four-wave mixing of topological edge plasmons in graphene metasurfaces

**DOI:** 10.1126/sciadv.aaz3910

**Published:** 2020-03-27

**Authors:** Jian Wei You, Zhihao Lan, Nicolae C. Panoiu

**Affiliations:** Department of Electronic and Electrical Engineering, University College London, Torrington Place, London WC1E 7JE, UK.

## Abstract

We study topologically protected four-wave mixing (FWM) interactions in a plasmonic metasurface consisting of a periodic array of nanoholes in a graphene sheet, which exhibits a wide topological bandgap at terahertz frequencies upon the breaking of time reversal symmetry by a static magnetic field. We demonstrate that due to the significant nonlinearity enhancement and large life time of graphene plasmons in specific configurations, a net gain of FWM interaction of plasmonic edge states located in the topological bandgap can be achieved with a pump power of less than 10 nW. In particular, we find that the effective nonlinear edge-waveguide coefficient is about γ ≃ 1.1 × 10^13^ W^−1^ m^−1^, i.e., more than 10 orders of magnitude larger than that of commonly used, highly nonlinear silicon photonic nanowires. These findings could pave a new way for developing ultralow-power-consumption, highly integrated, and robust active photonic systems at deep-subwavelength scale for applications in quantum communications and information processing.

## Teaser

It is demonstrated that net optical gain can be achieved via topologically protected four-wave mixing in a graphene metasurface.

## INTRODUCTION

In the past decade, topological photonics has emerged as a rapidly burgeoning field of exploration of topological physics ideas framed in the context of photonics. This area of research began with the theoretical work by Haldane and Raghu (*1*, *2*), where they constructed an analog of quantum Hall edge states in photonic crystals based on magneto-optical media and observed topological edge modes within the corresponding photonic bandgaps. Shortly afterward, an experimental realization and observation of such topological edge modes in a magneto-optical photonic crystal were reported in the microwave regime (*3*). Since then, there has been increasing interest in implementing in photonics topological states of matter and developing new ideas specific to topological photonics (*4*–*8*).

In addition to new perspectives brought in fundamental science, topological photonics also offers a broad array of potential applications for novel photonic devices, as its exotic features have already prompted the reexamination of some traditional views on light manipulation and propagation. For instance, reducing back reflection is a major challenge in optical waveguides, and in this context, the unidirectional topological waveguide (*9*–*11*) is an ideal light transport device in integrated photonics, as it could transmit light without backscattering even in the presence of inherent structural disorder. Moreover, some new concepts of topological photonics have also led to the development of novel photonic devices, such as optical isolators (*12*, *13*), robust delay lines (*14*, *15*), signal switches (*16*), nonreciprocal devices (*17*–*19*), and topological lasers (*20*, *21*).

Most of the previous studies have focused on linear topological photonic systems; however, topological physics could also play an important role in the nonlinear regime, leading to previously unknown collective phenomena and strongly correlated states of light (*8*, *22*–*24*). For instance, a topological source of quantum light has recently been realized in a nonlinear photonic system, which paves a new way for the development of robust quantum photonic devices (*22*). Moreover, a novel and sensitive approach for imaging topological edge states with third-harmonic generation has been recently demonstrated experimentally (*24*).

In this work, we demonstrate topologically protected four-wave mixing (FWM) interactions in a graphene metasurface upon the breaking of time reversal symmetry by a static magnetic field. In particular, we show that due to large optical near-field enhancement and large life time of graphene plasmons in such metasurfaces, a net gain of FWM interaction of plasmonic edge states located in the topological bandgap can be achieved at an ultralow pump power of less than 10 nW. This remarkable feature is a direct consequence of the unusually large effective nonlinear edge-waveguide coefficient, γ ≃ 1.1 × 10^13^ W^−1^ m^−1^, which, to the best of our knowledge, to date is by far the largest reported nonlinear optical coefficient of a light guiding physical system.

## RESULTS

### The system

We study a topologically protected nonlinear FWM process in a graphene plasmonic system. Graphene distinguishes itself as an ideal platform to study nonlinear topological photonics in several key aspects: First, graphene exhibits large nonlinearity over a broad spectral range, from terahertz to visible light. In particular, it has been shown (*25*) that graphene in a strong magnetic field has the largest third-order susceptibility of all known materials. Second, some recent studies (*26*, *27*) revealed that topologically protected one-wave edge plasmons can be realized in graphene metasurfaces. In addition to the typical plasmonic effects, such as local field enhancement and field confinement, the local field can be further confined to the edge of the graphene plasmonic system, leading to a marked enhancement of the nonlinear optical response of the graphene system. Third, phase matching is a crucial condition in nonlinear frequency mixing processes. In contrast to the frequently used bulk modes (*22*), where several modes with different wave vectors usually exist at a given frequency, a topological edge mode with absolute value of the Chern number equal to one, as it is the case with the graphene metasurface investigated in this study, has a unique wave vector at a fixed frequency. Therefore, the phase matching condition can be achieved and implemented experimentally much more easily, as in this case only one mode can be excited at a specific frequency. These important features make graphene plasmonic systems particularly appealing in the design of highly integrated nonlinear topological nanophotonic devices.

**Fig. 1:**
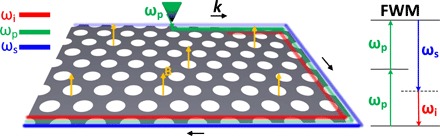
FWM of topologically protected one-way edge plasmons in a graphene metasurface consisting of a periodic nanohole array with hexagonal symmetry in a static magnetic field and the corresponding energy level diagram of a degenerate FWM process.

The nonlinear system explored in this work is illustrated in Fig. 1. A graphene plasmonic metasurface consisting of a periodic nanohole array with hexagonal symmetry is placed in a static magnetic field. Because of the time reversal symmetry breaking induced by the magneto-optical response of graphene under an external magnetic field, this plasmonic system could have a topological bandgap. After a geometry optimization, the topological bandgap could become wide enough so that it readily accommodates the optical modes taking part in an FWM process. To induce an FWM process, the system is excited by an external source at the pump frequency ω_p_, as illustrated in Fig. 1. Because of the strong third-order nonlinearity of graphene, a degenerate FWM process could take place, where two photons in a pump mode will generate a pair of photons at the signal and idler frequencies, ω_s_ and ω_i_, respectively. As a result, the energy of the pump mode (green) in Fig. 1 is transferred to the (seeded) signal (blue) and idler (red) modes, leading to the pump decay and the amplification of the signal and idler. This degenerate FWM process is topologically protected by the chiral nature of the edge plasmons.

### Topological bands of the graphene plasmonic system: Linear response

**Fig. 2:**
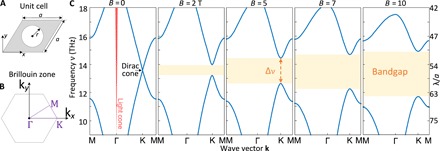
Band diagrams of the graphene plasmonic metasurface. (**A**) Unit cell and (**B**) the first Brillouin zone of the metasurface. (**C**) Band diagrams of the metasurface at *B* = 0, 2, 5, 7, 10 T. As the Dirac cone is below the air light cone, surface plasmons can exist at deep-subwavelength scale (λ/*a* > 40). Moreover, a topological bandgap is opened in the presence of an external static magnetic field.

We first study the linear optical response of our graphene plasmonic system by calculating its photonic band structure using a numerical approach based on the finite element method (FEM). The unit cell (with lattice constant *a* and air hole radius *r*) and the first Brillouin zone of the system used in our simulations are shown in Fig. 2 (A and B). The band diagrams of the system (*a* = 400 nm, *r* = 120 nm) at different magnetic fields *B* = 0,2,5,7,10 T are presented in Fig. 2C, where the parameters of the graphene are set to be *E*_F_ = 0.2 eV, *v*_F_ = 10^6^ ms^−1^, and τ = 50 ps (see Materials and Methods for details).

There are several notable features of the results presented in Fig. 2C. First, without the external magnetic field (*B* = 0), due to the hexagonal symmetry of the metasurface structure, Dirac cones that are protected by the parity (*P*) inversion and time reversal (*T*) symmetries exist at *K* and *K′* symmetry points of the Brillouin zone (*26*). Second, in the presence of the magnetic field (*B* ≠ 0), the time reversal symmetry of the system is broken and, consequently, the Dirac cones are gapped out, resulting in a topological nontrivial bandgap. Moreover, the width ∆*v* of this bandgap increases as the amplitude of the magnetic field increases.

**Fig. 3:**
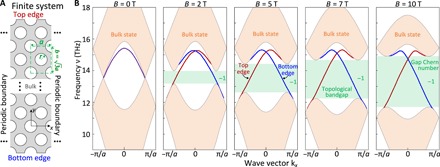
Band diagrams of a finite graphene metasurface. (**A**) Geometry of the finite graphene metasurface, where the number of unit cells (green dashed frame) is finite along the *y* axis and infinite along the *x* axis. (**B**) Projected band diagrams of the metasurface at *B* = 0, 2, 5, 7, and 10 T, where the edge modes on the top and bottom boundaries are depicted by red and blue curves, respectively. Since the gap Chern number characterizes the number of edge modes in the gap, there is a single edge mode at each boundary.

To confirm the topological nature of the bandgap, we illustrate the emergence of the edge modes within the bandgap for a system that has finite size along the *y* axis, i.e., the number of unit cells along this direction is finite (chosen to be 20 in our FEM simulations), whereas the system is periodic along the *x* axis (in the FEM simulations, periodic boundary conditions are imposed at the left and right boundaries along the *x* axis; see Fig. 3A). The supercell for this finite system is shown by a green rectangle in Fig. 3A, whose width and length are *a* and $b=\sqrt{3a}$, respectively.

The projected band diagrams along *k_x_*, determined for *B* = 0, 2, 5, 7, 10 T, are depicted in Fig. 3B. First, similar to what we observed in Fig. 2C, a bandgap opens when one applies an external static magnetic field (*B* ≠ 0), with the gap width ∆*v* increasing as the amplitude of magnetic field increases. However, different from the band diagrams of an infinite graphene metasurface shown in Fig. 2C, in the band diagrams of Fig. 3B, there are two additional edge modes at the top (red) and bottom (blue) boundaries of the finite graphene system. These two edge modes connect the bulk bands located above and below the bandgap and cannot be moved out of the bandgap into the bulk bands as long as the bandgap exists. In other words, they are robust and defect immune, which is guaranteed by the topological protection of the bandgap.

One can also calculate the gap Chern number, which is a topological invariant that characterizes the topological properties of the bandgap, to further confirm that the bandgap discussed above is topologically nontrivial and that these edge modes are topological modes (*26*). To this end, we indicate in Fig. 3B the calculated gap Chern number. Since the gap Chern number is −1 (the magnitude indicates the number of topological edge modes, whereas the sign shows the direction of propagation), there is only one topologically protected edge mode for each edge termination. In other words, our graphene structure supports modes that can exhibit unidirectional and defect-immune propagation features along the top and bottom edges. Moreover, the property of unidirectional propagation of the edge modes is also illustrated by the slope of their frequency dispersion curve, as their group velocity, *v*_g_ = ∂ω/∂*k*, within the topological bandgap is either positive (top edge) or negative (bottom edge).

**Fig. 4:**
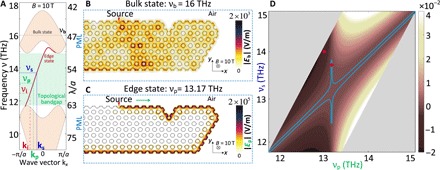
Nonlinear edge-mode interaction and FWM within a topological bandgap. (**A**) Band diagram of the graphene metasurface at *B* = 10 T. (**B**) Field profile of a bulk mode excitation at *v*_b_ = 16 THz, showing that the optical field spreads throughout the bulk region. (**C**) Field profile of an edge mode excitation at *v*_b_ = 13.17 THz, illustrating its unidirectional and defect-immune propagation along the system edge (τ → ∞). (**D**) Dispersion map of the normalized wave vector mismatch Δκ. The blue contour is defined by the condition Δκ = 10^−5^, whereas the red and magenta dots correspond to a nearly phase-matched (Δκ = 5.16 × 10^−6^) and a phase-mismatched (Δκ = 1.75 × 10^−2^) FWM process, respectively.

To gain deeper insights into the physical properties of plasmonic bulk and edge modes of the graphene metasurface, the near-field distribution of these modes propagating in a finite graphene plasmonic metasurface (four unit cells along the *y* axis and about 15 unit cells along the *x* axis, as per Fig. 4, B and C) is studied by using full-wave FEM simulations. In these computations, a perfectly matched layer is used at the left side of the graphene structure, whereas at the other sides, we imposed scattering boundary conditions so as to mimic infinite air space. To excite this finite graphene system, an electric source (*E*_0_ = 2 × 10^4^ V/m) depicted by a red triangle in Fig. 4B is used.

In the case of the bulk mode, we choose the source frequency *V*_b_ = 16 THz, which belongs to the bulk region (see Fig. 4A). As expected, the corresponding optical field spreads throughout the graphene structure (see Fig. 4B), which proves that bulk modes are excited in this case. It should be noted that, due to the plasmonic characteristics of the graphene bulk modes, their optical field is tightly confined at the surface of the graphene metasurface.

In the case of the excitation of an edge mode, we choose a frequency in the bandgap, *v*_p_ = 13.17 THz, so that at this frequency only the edge mode exists, as per Fig. 4A. The corresponding field profile generated by the source in the finite system, presented in Fig. 4C, illustrates several notable features. Thus, the optical field does not penetrate in the bulk region and only propagates unidirectionally along the edge of the graphene metasurface. In addition, because of the chiral nature of the edge mode, this unidirectional propagation is robust against structural defects, which allows it to circumvent defects (e.g., sharp bends) without producing backscattering. These features prove that the edge modes within the topological bandgap in Fig. 4A are topologically protected. Last but not least, we note that in addition to the plasmonic field confinement effect illustrated in Fig. 4 (B and C), the optical field of the edge modes is further confined to the edge of the system, which is particularly important when one seeks to enhance nonlinear optical interactions such as FWM.

### Nonlinear interaction of edge states: FWM

Comparing Fig. 4 (B and C), one can see that, in the case of the edge mode, the plasmon-induced field enhancement effect quantified by the ratio ∣*E*_e_∣_max_/*E*_0_ is two orders of magnitude stronger than that in the case of the bulk mode ∣*E*_b_∣_max_/*E*_0_, namely, ∣*E*_e_∣_max_/∣*E*_b_∣_max_ > 100. Moreover, the results in Fig. 4A show that at a particular frequency, there is only a single-edge mode, with a unique wave vector, whereas several different bulk modes can be excited at one frequency. The former effect is important in enhancing the nonlinear optical interactions, whereas the latter one is particularly useful for engineering physical configurations in which phase matching in FWM is achieved.

To illustrate these ideas, in what follows, we analyze the circumstances in which the phase matching condition in a degenerate FWM of one-way edge modes can be fulfilled. To this end, we calculate the normalized wave vector mismatch, defined as Δκ ≡ αΔk = *a*(2*k*_p_ − *k*_s_ − *k_i_*), corresponding to an FWM process in which a pump edge mode with wave vector **k**_p_ gives rise to signal and idler edge modes with wave vectors **k**_s_ and **k**_i_, respectively. In particular, the exchange of energy among the interacting waves is most efficient when the phase matching condition ∆κ = 0 is satisfied. Unlike the wave vector, the energy is conserved in the FWM process, meaning 2*v*_p_ = *v*_s_ + *v*_i_.

Starting from the mode dispersion curves of the topological edge modes presented in Fig. 4A, and using the energy conservation relation that characterizes the FWM process, the corresponding dispersion map of the normalized wave vector mismatch Δκ is calculated numerically and depicted in Fig. 4D. In particular, for the sake of a better quantitative understanding of the energy conversion efficiency of the FWM process, we also show in this figure the contour defined by Δκ = 10^−5^. More specifically, for frequencies inside the domain defined by this contour, energy is transferred from the pump to the signal and idler over a distance of about 10^5^π lattice constants.

To validate the conclusions derived from the dispersion map of Δκ, we performed full-wave simulations of the nonlinear dynamics of the interacting edge modes. To this end, we chose a point indicated with a red dot in Fig. 4D, characterized by *v*_p_ = 13.17 THz, *v*_s_ = 13.72 THz, and *v*_i_ = 12.62 THz, and for which the FWM interaction is nearly phase matched (Δκ = 5.16 × 10^−6^). Moreover, we considered a seeded FWM process in which the input intensity of the signal is much smaller than that of the pump, whereas the input intensity of the idler is set to zero. Specifically, in our FEM simulations, we set the source input field amplitudes at the three frequencies ∣*E*_p_∣ = 2 × 10^4^ V m^−1^, ∣*E*_s_∣ = 4 × 10^2^ V m^−1^, and ∣*E*_i_∣ = 0. Last, the nonlinearity of graphene under the influence of a magnetic field of 10 T is described by a third-order susceptibility with value of χ^(3)^ = 5 × 10^−10^ m^2^ V^2^ (*25*).

**Fig. 5:**
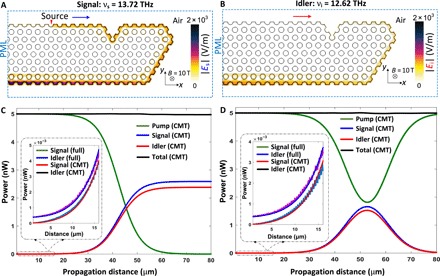
Topologically protected FWM process in a graphene metasurface. (**A**) Field profile at the signal frequency, *v*_s_ = 13.72 THz. (**B**) Field profile at the idler frequency, *v*_i_ = 16.62 THz. (**C**) Dependence on the propagation distance of the mode power of the pump, signal, and idler corresponding to the field profiles shown in (A) and (B), determined using the CMT when the FWM process is phase matched. Also shown in the insets are the same mode powers determined using the CMT and full-wave simulations. The black curve corresponds to the total power and shows that the energy is conserved in the FWM interaction. (**D**) The same as in (C), but corresponding to a case when the FWM interaction is not phase matched.

Using the procedure we just described, we have computed the near-field profiles at the frequencies of the pump, signal, and idler, the results of these simulations being summarized in Fig. 5. In these calculations, graphene losses are neglected by setting τ → ∞. It can be seen in Fig. 5 (A and B) that, as a result of the nonlinear FWM interaction, the signal is amplified upon propagation, whereas an edge mode is generated at the idler frequency (note that in Fig. 5B, there is no external source at the idler frequency). Since the frequency of all the interacting edge modes is located in the topological bandgap, both signal and idler modes are topologically protected and exhibit unidirectional and defect-immune propagation along the system edge.

The FWM process can be further quantitatively investigated by calculating the dependence on the propagation distance of the power carried by the three edge modes. The mode power is calculated by integrating the corresponding Poynting vector across the transverse section of the mode. The results of these calculations are summarized in Fig. 5 (C and D) and correspond to the case of near phase matching discussed above and a case when the FWM process is not phase matched. In the latter case, the system parameters are *v*_p_ = 13.03 THz, *v*_s_ = 14.05 THz, *v*_i_ = 12.01 THz, and ∆κ = 1.75 × 10^−2^ (magenta point in Fig. 4D).

There are several important ideas revealed by the results presented in Fig. 5 (C and D). First, the power of both the signal and idler modes is amplified upon propagation, due to the energy conversion from the pump mode. Second, the growth rate of the signal and idler modes in the case of the nearly phase-matched FWM is larger than when the FWM interaction is not phase matched, which means that the energy conversion is more efficient in the former case. Third, the plots presented in the insets of Fig. 5 (C and D) show that the predictions of the coupled-mode theory (CMT), see the Supplementary Materials for details, agree very well with the rigorous results obtained using full-wave simulations of the nonlinear mode interaction, despite the fact that the optical fields at the three frequencies are strongly confined at deep-subwavelength scale and significantly enhanced. This is particularly important in practice because the CMT calculations are much faster and require a much smaller amount of memory as compared with using full-wave simulations.

Our CMT predicts that the effective nonlinear FWM coefficient is γ_FWM_ ≈ 2.4 × 10^13^ W^−1^ m^−1^ (see the Supplementary Materials), which is more than 10 orders of magnitude larger than that of silicon photonic wire waveguides (*28*, *29*) and 5 orders of magnitude larger than that of a graphene nanoribbon waveguide (*30*). To the best of our knowledge, to date, this is the largest nonlinear FWM coefficient reported in a nonlinear optical system. This remarkable result is a consequence of the particularly large third-order susceptibility of graphene, which is further enhanced by the plasmon-induced enhancement and extreme confinement of the optical field of the edge modes. In particular, the size of the unit cell of the graphene metasurface is much smaller than the operating wavelength, namely, λ/*a* > 50 in our FWM process, a notable feature that can facilitate the design of low-power, ultracompact active photonic nanodevices.

**Fig. 6:**
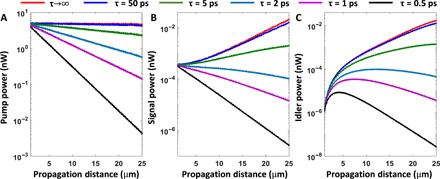
Influence of loss on the topologically protected FWM process in graphene metasurface at *B* = 10 T. (**A** to **C**) Dependence on the propagation distance of the power of the pump, signal, and idler edge modes, respectively, corresponding to a phase-matched FWM process, determined for different values of the loss rate. Because of the particularly large value of the FWM coefficient, net gain (the FWM gain overcompensates the loss) can be achieved as long as τ ≳ 2.5 ps.

The radiation loss of the edge modes of the graphene metasurface can be neglected because, as we just discussed, they are strongly confined. The intrinsic loss, however, has to be taken into account in practical applications. To study its influence on the FWM interaction, a finite plasmon life time τ is considered in Eqs. 2 and 3. Similar to the lossless case, we determined the dependence of the power of the interacting edge modes on the propagation distance; the corresponding results of this analysis are presented in Fig. 6.

Typically, the plasmon lifetime is determined by the plasmon-phonon coupling and varies from 0.1 to 1 ps (*31*). This loss can be reduced if exfoliated graphene is placed onto a boron nitride substrate (*32*), which leads to a lifetime as large as 3 ps. Moreover, recent experiments (*33*, *34*) have demonstrated that an external magnetic field can also strongly increase the plasmon lifetime, as in this case the two-dimensional surface plasmon can be effectively transformed into a one-dimensional–like edge plasmon. Specifically, it has been shown that by applying an external magnetic field, the plasmon lifetime can be readily increased to 50 ps (*34*).

One remarkable conclusion of the analysis of the FWM interaction of edge modes of our lossy graphene metasurface is that net signal gain can be achieved if the lifetime τ ≳ 2.5 ps. This is the first plasmonic system in which net gain can be achieved without incorporating gain optical media in the system. It can be seen in Fig. 6 that whereas the pump decays for all values of the plasmon lifetime, due to the combined contributions of the graphene loss and energy transfer mediated by the FWM interaction, the signal power increases monotonously if τ ≳ 2.5 ps. The idler, on the other hand, shows a more complex dynamics. Thus, irrespective of the value of the life time, at the beginning of the nonlinear interaction, the power in the idler builds up over a certain distance. After this amplification stage, the power in the idler decays monotonously if τ ≲ 2.5 ps, because the pump power is no longer large enough to sustain the amplification of the idler, whereas if τ ≳ 2.5 ps, then the power in the idler mode increases monotonously over the entire distance considered in our simulations.

## DISCUSSION

Using rigorous full-wave simulations supported by a coupled-mode theory, we have demonstrated a topologically protected nonlinear FWM process in a patterned graphene plasmonic metasurface. In particular, we have shown that a topological bandgap as wide as several terahertz can be created in the metasurface under a strong static magnetic field. Moreover, the analysis of the dispersion properties of the topologically protected edge modes located in the bandgap reveals that FWM interaction is efficiently phase matched in a large domain of the parameter space of the system. Here, we note the dispersion property of edge modes can be engineered by tailoring the edge truncation of the graphene metasurface. The near-field profiles of the interacting edge modes show unidirectional and defect-immune propagation, hence demonstrating that the FWM process is topologically protected. Our study also reveals that, because of an unusually large value of the FWM nonlinear coefficient and the large field enhancement at plasmon resonances, the FWM interaction produces net gain even when plasmon losses in graphene are rigorously taken into account. This notable property of FWM of topological edge modes of graphene metasurfaces might play an important role in the development of new ultracompact and topologically protected active photonic nanodevices.

## MATERIALS AND METHODS

In our modeling of an infinite graphene metasurface, periodic boundary conditions are used for the four edges of the unit cell depicted in Fig. 2A. At infrared and terahertz frequencies, graphene placed in a static magnetic field can be characterized as an electrically gyrotropic material (*35*–*37*), whose surface conductivity tensor can be represented as

$$\sigma_{s}=[\begin{array}{cc} \sigma_{L} & \sigma_{H}\\ -\sigma_{H} & \sigma_{L} \end{array}]$$ (1)

where the diagonal elements (longitudinal conductivity, σ_L_) and the off-diagonal elements (Hall conductivity, σ_H_) can be determined using Kubo’s formalism (*38*). At room temperature and for frequencies below the visible-light region, the longitudinal and Hall conductivities are given by (*26*, *27*)

$$\sigma_{L}=\sigma_{0}\frac{1-i\omega \tau }{{\left(\omega_{c}\tau \right)}^{2}-{\left(i+\omega \tau \right)}^{2}}$$ (2)

$$\sigma_{H}=-\sigma_{0}\frac{\omega_{c}\tau }{{\left(\omega_{c}\tau \right)}^{2}-{\left(i+\omega \tau \right)}^{2}}$$ (3)

where σ_0_ = *e*^2^*E*_F_τ/(πℏ^2^), τ is the relaxation time (plasmon lifetime), $\omega_{c}\approx eB_{⊥}v_{F}^{2}/E_{F}$ is the cyclotron frequency, with *B*_⊥_, *v*_F_, and *E*_F_ being the external static magnetic field perpendicular onto the graphene surface, the graphene Fermi velocity, and the Fermi energy, respectively.

The surface conductivities in Eqs. 2 and 3 show that graphene under a nonzero static magnetic field is lossy; thus, the eigenvalue problem defining the band structure of graphene metasurfaces becomes non-Hermitian. Therefore, most of the traditional electromagnetic eigenmode solvers are not particularly efficient to compute band diagrams of such metasurfaces. To circumvent this problem, we used the numerical solver of Comsol based on the FEM method to calculate the band diagrams of the graphene metasurfaces investigated in this study. In our full-wave simulations, electric sources are placed at points with low symmetry, so that all modes are excited. Moreover, multiple probe monitors are placed at low-symmetry points, too, to determine the mode frequencies.

In the nonlinear simulations, three nonlinear surface currents are defined in the Comsol software, namely, one for the pump frequency (*v*_p_), one for the signal frequency (*v*_s_), and one for the idler frequency (*v*_i_). These nonlinear currents are coupled via the optical fields of the interacting modes, as described by the following equations

$$J_{p}^{\text{surf}}=6\sigma_{p,\text{surf}}^{\left(3\right)}E_{s}E_{i}E_{p}^{*}$$ (4)

$$J_{s}^{\text{surf}}=3\sigma_{s,\text{surf}}^{\left(3\right)}E_{p}E_{p}E_{i}^{*}$$ (5)

$$J_{i}^{\text{surf}}=3\sigma_{i,\text{surf}}^{\left(3\right)}E_{p}E_{p}E_{s}^{*}$$ (6)

Here, the third-order surface conductivity is defined as $\sigma_{\alpha ,\text{surf}}^{\left(3\right)}=-i\epsilon_{0}\omega_{\alpha }h_{\text{eff}}\chi^{\left(3\right)}$, α = p, s, i, where χ^(3)^ is the third-order bulk susceptibility and the thickness of graphene is assumed to be *h*_eff_ = 0.3 nm (*25*). Moreover, the electric fields *E*_α_, α = p, s, i, are the amplitudes of the pump, signal, and idler, respectively. More details about the nonlinearity of magnetized graphene and the coupled-mode theory describing the FWM interaction of graphene edge modes can be found in the Supplementary Materials.

## Supplementary Material

aaz3910_SM.pdf

## SUPPLEMENTARY MATERIALS

Supplementary material for this article is available at http://advances.sciencemag.org/cgi/content/full/6/13/eaaz3910/DC1 Section S1. Optical properties of graphene in an external static magnetic field Section S1.1. Linear optical properties of graphene Section S1.2. Nonlinear optical properties of graphene Section S2. Coupled-mode theory describing the FWM nonlinear process Section S3. Influence of optical losses on graphene topological plasmonic systems Section S4. Influence of the substrate on graphene topological plasmonic systems Fig. S1. Dependence of the effective waveguide nonlinear coefficient γ on *z*. Fig. S2. Effect of optical losses as described by the coupled-mode theory. Fig. S3. Band diagrams of a graphene metasurface on a PMMA substrate. References (*39*–*45*)
