# Supplementary material for: Four-wave mixing of topological edge plasmons in graphene metasurfaces
Source: Sci Adv. 2020 Mar 27;6(13):eaaz3910. doi: 10.1126/sciadv.aaz3910 (PMC7101229; doi:10.1126/sciadv.aaz3910)
Supplement: aaz3910_SM.pdf [file aaz3910_SM.pdf]

Science Advances
AAAS

advances.sciencemag.org/cgi/content/full/6/13/eaaz3910/DC1

Supplementary Materials for

Four-wave mixing of topological edge plasmons in graphene metasurfaces

Jian Wei You, Zhihao Lan, Nicolae C. Panoiu*

*Corresponding author. Email: n.panoiu@ucl.ac.uk

Published 27 March 2020, *Sci. Adv.* **6**, eaaz3910 (2020)
DOI: 10.1126/sciadv.aaz3910

This PDF file includes:

Section S1. Optical properties of graphene in an external static magnetic field
Section S1.1. Linear optical properties of graphene
Section S1.2. Nonlinear optical properties of graphene
Section S2. Coupled-mode theory describing the FWM nonlinear process
Section S3. Influence of optical losses on graphene topological plasmonic systems
Section S4. Influence of the substrate on graphene topological plasmonic systems
Fig. S1. Dependence of the effective waveguide nonlinear coefficient γ on z.
Fig. S2. Effect of optical losses as described by the coupled-mode theory.
Fig. S3. Band diagrams of a graphene metasurface on a PMMA substrate.
References (*39–45*)

Section S1. Optical properties of graphene in an external static magnetic field

Section S1.1. Linear optical properties of graphene

At infrared and terahertz frequencies, graphene placed in a static magnetic field can be characterized as an electrically gyrotropic material (*35-37*), whose surface conductivity tensor is given by

$$\boldsymbol{\sigma_s} = \begin{pmatrix} \sigma_L & \sigma_H & 0 \\ -\sigma_H & \sigma_L & 0 \\ 0 & 0 & 0 \end{pmatrix}, \tag{S1}$$

where the diagonal element (longitudinal conductivity, σ_L) and the off-diagonal element (Hall conductivity, σ_H) are determined using Kubo's formalism (*38*). At room temperature and for frequencies below the visible-light region, the longitudinal and Hall conductivities are given by (*26,27*):

$$\sigma_L = \sigma_0 \frac{\tau^{-1} - i\omega}{{\omega_c}^2 - (\omega + i\tau^{-1})^2}, \tag{S2a}$$

$$\sigma_H = -\sigma_0 \frac{\omega_c}{{\omega_c}^2 - (\omega + i\tau^{-1})^2}, \tag{S2b}$$

where $\sigma_0 = e^2 E_F \tau / (\pi \hbar^2)$, τ is the relaxation time (plasmon lifetime), $\omega_c \approx eB_\perp v_F^2 / E_F$ is the cyclotron frequency, with $B_\perp$, v_F, and E_F being the external static magnetic field perpendicular onto the graphene surface, the graphene Fermi velocity, and the Fermi energy, respectively. The relationship between the bulk permittivity and surface conductivity is given by $\boldsymbol{\epsilon}_r = \mathbf{I}_3 + i\boldsymbol{\sigma_s}/(\epsilon_0 \omega h_{eff})$, where $\mathbf{I}_3$ is the 3×3 identity matrix and h_{eff} is the effective thickness of monolayer graphene. In order to simplify the derivation of the following coupled-mode equations (CMEs), the permittivity tensor $\boldsymbol{\epsilon}_r$ is recast as a combination of a Hermitian part $\boldsymbol{\epsilon}_H$ and a non-Hermitian part $\boldsymbol{\epsilon}_{NH}$, namely $\boldsymbol{\epsilon}_r = \boldsymbol{\epsilon}_H + \boldsymbol{\epsilon}_{NH}$, where

$$\boldsymbol{\epsilon}_H = \frac{1}{2}(\boldsymbol{\epsilon}_r + \boldsymbol{\epsilon}_r^\dagger), \tag{S3a}$$

$$\boldsymbol{\epsilon}_{NH} = \frac{1}{2}(\boldsymbol{\epsilon}_r - \boldsymbol{\epsilon}_r^\dagger). \tag{S3b}$$

Here, the symbol "†" denotes the Hermitian transpose operation. In this way, the Hermitian part could be conveniently considered as the lossless contribution of graphene to the following derivation of CMEs, whereas the non-Hermitian term is treated as the loss contribution. Since the intrinsic loss of graphene is very small in the infrared regime, the loss effect of graphene can be considered as a perturbation term in the coupled-mode theory (CMT).

Section S1.2. Nonlinear optical properties of graphene

The crystal structure of graphene has the symmetry of the D_{6h} point group, thus the first nonvanishing nonlinear susceptibility of graphene is the third-order susceptibility $\boldsymbol{\chi}^{(3)}$. Recently, theoretical and experimental works (*25,39,40*) have suggested that the third-order susceptibility $\boldsymbol{\chi}^{(3)}$ of graphene is much stronger than that of most optical materials. For instance, in (*39,40*), it has been shown experimentally that the third-order susceptibility of graphene is about $10^{-15}\,\mathrm{m}^2\,\mathrm{V}^{-2}$, which is almost two orders of magnitude larger than that of gold films and eight orders of magnitude larger than that of most insulator materials, such as glass and CaF_2. More importantly, it has been demonstrated (*25*) that the third-order susceptibility of graphene under a strong magnetic field can be further enhanced. For instance, when the external magnetic field is about $5\,\mathrm{T}$, the predicted effective bulk third-order susceptibility of graphene is $\chi^{(3)} = 10^{-9}\,\mathrm{m}^2\,\mathrm{V}^{-2}$, which is almost six orders of magnitude larger than that of unmagnetized graphene. In (*25*), the effective bulk third-order susceptibility of magnetized graphene is approximated as $\chi^{(3)} = 5\times 10^{-9}\,\mathrm{m}^2\,\mathrm{V}^{-2}/[B(T)]$, where the magnetic field $B(T)$ is expressed in Tesla.

Section S2. Coupled-mode theory describing the FWM nonlinear process

The permittivity tensor $\boldsymbol{\epsilon}_r$ of graphene under an external static magnetic field is no longer a Hermitian matrix. However, since the intrinsic loss of graphene, described by the non-Hermitian part of the permittivity tensor $\boldsymbol{\epsilon}_{NH}$, is relatively weak in the infrared regime, it can be treated as a perturbation in the CMT. As a result, in order to derive the CME describing the four-wave mixing (FWM) of topological plasmon modes o four graphene metasurface, we employ the conjugated form of the Lorentz reciprocity theorem (*41-44*) with graphene described by the Hermitian part of the permittivity tensor, $\boldsymbol{\epsilon}_H$. To this end, let us consider two solutions of Maxwell equations, $\{\mathbf{E}_a(\mathbf{r}),\ \mathbf{H}_a(\mathbf{r})\}$ and $\{\mathbf{E}_b(\mathbf{r}),\ \mathbf{H}_b(\mathbf{r})\}$, which correspond to the perturbed and unperturbed systems, respectively. More specifically, the unperturbed system consists of the graphene metasurface for which the loss and optical nonlinearity vanish, whereas the perturbed system corresponds to the case in which these two effects are taken into account.

For the unperturbed system, the Maxwell equations are written as:

$$\nabla\times\mathbf{E}_a(\mathbf{r},\omega) = i\omega\mu\mathbf{H}_a(\mathbf{r},\omega), \tag{S4a}$$
$$\nabla\times\mathbf{H}_a(\mathbf{r},\omega) = -i\omega\boldsymbol{\epsilon}_a(\mathbf{r},\omega)\mathbf{E}_a(\mathbf{r},\omega), \tag{S4b}$$

with

$$\mathbf{E}_a(\mathbf{r},\omega_m) = \frac{\mathbf{e}_m(\mathbf{r},\omega_m)}{\sqrt{P_m}}e^{i\beta_m z}, \tag{S5a}$$
$$\mathbf{H}_a(\mathbf{r},\omega_m) = \frac{\mathbf{h}_m(\mathbf{r},\omega_m)}{\sqrt{P_m}}e^{i\beta_m z}, \tag{S5b}$$

where ω_m and $\beta_m = \beta(\omega_m)$, $m = 1,2,3,4$, are the frequencies of the interacting modes involved in the FWM nonlinear process and the corresponding propagation constants, respectively, $\mathbf{e}_m(\mathbf{r},\omega_m)$ and $\mathbf{h}_m(\mathbf{r},\omega_m)$ are the modal fields, $\mu = \mu_0$ is the vacuum permeability, $\boldsymbol{\epsilon}_a(\mathbf{r},\omega) = \boldsymbol{\epsilon}_H$ ($\boldsymbol{\epsilon}_a(\mathbf{r},\omega) = \epsilon_0$) if $\mathbf{r}$ belongs to the graphene (air) regions, and we assume the modes propagate along the z-direction. If we choose the normalization constants P_m such that (*28,45*):

$$\frac{1}{4}\int_S (\mathbf{e}_m\times\mathbf{h}^*_{m'} + \mathbf{e}^*_{m'}\times\mathbf{h}_m)\cdot\mathbf{z}dS = P_m\delta_{mm'}, \tag{S6}$$

the modes defined in Eqs. (S5) carry a power of $1\,\mathrm{W}$.

The mode power is related to the mode energy contained in one unit cell via the following relation:

$$P = W\frac{v_g}{a} = \left(W^E + W^H\right)\frac{v_g}{a}, \tag{S7}$$

where a is the lattice constant and v_g is the group velocity of the optical mode. The definitions of electric and magnetic energies, W^E and W^H, respectively, are given by:

$$W = W^E + W^H = \frac{1}{4}\int_{V_{cell}}\left\{\frac{\partial}{\partial\omega}\left[\omega\mathbf{e}(\mathbf{r},\omega)\cdot\boldsymbol{\epsilon}^*(\mathbf{r},\omega)\mathbf{e}^*(\mathbf{r},\omega)\right] + \mu|\mathbf{h}(\mathbf{r},\omega)|^2\right\}dV. \tag{S8}$$

Here, V_{cell} is the volume of the unit cell.

In the case of the perturbed system, the perturbation of the system is described by the polarization $\mathbf{P}_{pert}(\mathbf{r},\omega)$, which is introduced in the Maxwell equations as:

$$\nabla \times \mathbf{E}_b(\mathbf{r},\omega) = i\omega\mu\mathbf{H}_b(\mathbf{r},\omega), \tag{S9a}$$

$$\nabla \times \mathbf{H}_b(\mathbf{r},\omega) = -i\omega\boldsymbol{\epsilon}_b(\mathbf{r},\omega)\mathbf{E}_b(\mathbf{r},\omega) - i\omega\mathbf{P}_{pert}(\mathbf{r},\omega), \tag{S9b}$$

where

$$\mathbf{E}_b = \sum_{n=1}^{4} A_n(z,\omega)\frac{\mathbf{e}_n(\mathbf{r},\omega_n)}{\sqrt{P_n}}e^{i\beta_n z}, \tag{S10a}$$

$$\mathbf{H}_b = \sum_{n=1}^{4} A_n(z,\omega)\frac{\mathbf{h}_n(\mathbf{r},\omega)}{\sqrt{P_n}}e^{i\beta_n z}. \tag{S10b}$$

The mode amplitudes $A_n(z,\omega_n)$, $n = 1,2,3,4$, describe the envelopes of the optical modes at frequency ω_n and are measured in units of $\sqrt{W}$.

In order to use the conjugated form of the Lorentz reciprocity theorem, we introduce the vector field:

$$\mathbf{F}_{ab} = \mathbf{E}_b \times \mathbf{H}_a^* + \mathbf{E}_a^* \times \mathbf{H}_b. \tag{S11}$$

Using the vector relation, $\nabla\cdot(\mathbf{A}\times\mathbf{B}) = \mathbf{B}\cdot(\nabla\times\mathbf{A}) - \mathbf{A}\cdot(\nabla\times\mathbf{B})$, one can easily show that

$$\nabla\cdot\mathbf{F}_{ab} = \mathbf{H}_a^*\cdot(\nabla\times\mathbf{E}_b) - \mathbf{E}_b\cdot(\nabla\times\mathbf{H}_a^*) + \mathbf{H}_b\cdot(\nabla\times\mathbf{E}_a^*) - \mathbf{E}_a^*\cdot(\nabla\times\mathbf{H}_b). \tag{S12}$$

Moreover, using the divergence theorem, it can be demonstrated that

$$\int_{S_\infty} \nabla\cdot\mathbf{F}_{ab}dS = \frac{\partial}{\partial z}\int_{S_\infty}\mathbf{F}_{ab}\cdot\mathbf{z}dS + \oint_{\partial S_\infty}\mathbf{F}_{ab}\cdot\hat{\mathbf{n}}dl, \tag{S13}$$

where S_∞ is the transverse section extending to infinity and perpendicular to the mode propagation direction (z-axis), ∂S_∞ is the boundary of S_∞, and $\hat{\mathbf{n}}$ is the unit vector outwardly normal onto the boundary ∂S_∞. Since the interacting modes are guided modes which decay exponentially when $\mathbf{r}\to\infty$, the last term in Eq. (S13) is equal to zero. Consequently, Eq. (S13) can be recast to:

$$\frac{\partial}{\partial z}\int_{S_\infty}\mathbf{F}_{ab}\cdot\mathbf{z}dS = \int_{S_\infty}\nabla\cdot\mathbf{F}_{ab}dS. \tag{S14}$$

Substituting Eqs. (S11) and Eqs. (S12) into Eq. (S14) and using Maxwell equations, namely Eqs. (S4) and Eqs. (S9), we obtain:

$$\begin{aligned}\frac{\partial}{\partial z}\int_{S_\infty}(\mathbf{E}_b\times\mathbf{H}_a^* + \mathbf{E}_a^*\times\mathbf{H}_b)\cdot\mathbf{z}dS =& i\int_{S_\infty}\left\{[\omega\mathbf{E}_a^*\cdot\boldsymbol{\epsilon}_b\mathbf{E}_b - \omega_m\mathbf{E}_b\cdot(\boldsymbol{\epsilon}_a\mathbf{E}_a)^*] + \mu(\omega-\omega_m)\mathbf{H}_a^*\cdot\mathbf{H}_b\right\}dS\\ &+ i\omega\int_{S_\infty}\mathbf{E}_a^*\cdot\mathbf{P}_{pert}dS, \quad m = 1,2,3,4.\end{aligned} \tag{S15}$$

For the l.h.s. of this equation, we use Eqs. (S5) and Eqs. (S10) to get:

$$\begin{aligned}\frac{\partial}{\partial z}\int_{S_\infty}(\mathbf{E}_b\times\mathbf{H}_a^* + \mathbf{E}_a^*\times\mathbf{H}_b)\cdot\mathbf{z}dS =& \frac{\partial}{\partial z}\sum_{n=1}^{4}\frac{A_n e^{i(\beta_n-\beta_m)z}}{\sqrt{P_m P_n}}\int_{S_\infty}(\mathbf{e}_n\times\mathbf{h}_m^* + \mathbf{e}_m^*\times\mathbf{h}_n)\cdot\mathbf{z}dS\\ =& 4\frac{dA_m}{dz} + \sum_{\substack{n=1\\ n\neq m}}^{4} C_{mn}\left[\frac{dA_n}{dz} + i(\beta_n-\beta_m)A_n\right], \quad m = 1,2,3,4,\end{aligned} \tag{S16}$$

where

$$C_{mn} = \frac{e^{i(\beta_n-\beta_m)z}}{\sqrt{P_m P_n}}\int_{S_\infty}(\mathbf{e}_n\times\mathbf{h}_m^* + \mathbf{e}_m^*\times\mathbf{h}_n)\cdot\mathbf{z}dS.$$

We now insert Eqs. (S5) and Eqs. (S10) into the r.h.s. of Eq. (S14), to obtain:

$$\begin{aligned}\int_{S_\infty} \nabla\cdot\mathbf{F}_{ab}dS =& i\mu(\omega-\omega_m)\int_{S_\infty}\mathbf{H}_a^*\cdot\mathbf{H}_b dS + i\int_{S_\infty}\left[\omega\mathbf{E}_a^*\cdot\boldsymbol{\epsilon}_b\mathbf{E}_b - \omega_m\mathbf{E}_b\cdot(\boldsymbol{\epsilon}_a\mathbf{E}_a)^*\right]dS + i\omega\int_{S_\infty}\mathbf{E}_a^*\cdot\mathbf{P}_{pert}dS \\ =& i\sum_{n=1}^{4}\frac{A_n e^{i(\beta_n-\beta_m)z}}{\sqrt{P_m P_n}}\left\{\int_{S_\infty}\left[\omega\mathbf{e}_m^*\cdot\boldsymbol{\epsilon}_b\mathbf{e}_n - \omega_m\mathbf{e}_n\cdot(\boldsymbol{\epsilon}_a\mathbf{e}_m)^*\right]dS + \mu(\omega-\omega_m)\int_{S_\infty}\mathbf{h}_m^*\cdot\mathbf{h}_n dS\right\} \\ &+ i\frac{\omega e^{-i\beta_m z}}{\sqrt{P_m}}\int_{S_\infty}\mathbf{e}_m^*\cdot\mathbf{P}_{pert}dS, \quad m=1,2,3,4. \end{aligned} \tag{S17}$$

This equation can be recast as:

$$\int_{S_\infty}\nabla\cdot\mathbf{F}_{ab}dS = \sum_{n=1}^{4}A_n D_{mn} + \frac{i\omega_n e^{-i\beta_m z}}{\sqrt{P_m}}\int_{S_\infty}\mathbf{e}_m^*\cdot\mathbf{P}_{pert}dS, \quad m=1,2,3,4, \tag{S18}$$

where

$$D_{mn}(\omega) = \frac{ie^{i(\beta_n-\beta_m)z}}{\sqrt{P_m P_n}}\int_{S_\infty}\left\{\mu(\omega-\omega_m)\mathbf{h}_m^*\cdot\mathbf{h}_n + \omega\mathbf{e}_m^*\cdot\boldsymbol{\epsilon}_b\mathbf{e}_n - \omega_m\mathbf{e}_n\cdot(\boldsymbol{\epsilon}_a\mathbf{e}_m)^*\right\}dS. \tag{S19}$$

Since the waveguide modes are mutually orthogonal, $D_{mn}(\omega_n)|_{n\neq m} = 0$. For the case when $n = m$, we have:

$$D_{mm}(\omega_m) = \frac{i\omega_m}{P_m}\int_{S_\infty}\left[\mathbf{e}_m^*\cdot\boldsymbol{\epsilon}_b\mathbf{e}_m - \mathbf{e}_m\cdot(\boldsymbol{\epsilon}_a\mathbf{e}_m)^*\right]dS. \tag{S20}$$

The permittivity tensor of lossless graphene in Eq. (S19) is a Hermitian matrix, and therefore $\int_{S_\infty}\left[\mathbf{e}\cdot(\boldsymbol{\epsilon}\mathbf{e})^* - \mathbf{e}^*\cdot\boldsymbol{\epsilon}\mathbf{e}\right]dS = 0$. This implies that $D_{mn} = 0$ if $n = m$, too.

Combining Eqs. (S14), (S16), and (S18) we obtain:

$$\frac{dA_m}{dz} + \sum_{\substack{n=1\\ n\neq m}}^{4}\frac{C_{mn}}{4}\left[\frac{dA_n}{dz} + i\left(\beta_n-\beta_m\right)A_n\right] = \frac{i\omega e^{-i\beta_m z}}{4\sqrt{P_m}}\int_{S_\infty}\mathbf{e}_m^*\cdot\mathbf{P}_{pert}dS, \quad m=1,2,3,4. \tag{S21}$$

In this equation, the perturbation $\mathbf{P}_{pert}$ describes both the linear contribution from the intrinsic loss of graphene and from the nonlinear optical effects. Therefore, we can write:

$$\mathbf{P}_{pert} = \mathbf{P}_{lin} + \mathbf{P}_{nl}. \tag{S22}$$

The linear polarization describes the influence of optical loss in graphene on the mode dynamics and can be written as:

$$\mathbf{P}_{lin} = \epsilon_0\boldsymbol{\epsilon}_{NH}\mathbf{E}_b = \epsilon_0\boldsymbol{\epsilon}_{NH}\sum_{n=1}^{4}A_n(z,\omega)\frac{\mathbf{e}_n(\mathbf{r},\omega_n)}{\sqrt{P_n}}e^{i\beta_n z}. \tag{S23}$$

The nonlinear contribution to $\mathbf{P}_{pert}$ is described by a third-order nonlinear susceptibility, $\boldsymbol{\chi}^{(3)}(\mathbf{r})$, so that $\mathbf{P}_{nl}$ can be expressed as:

$$\mathbf{P}_{nl} = \epsilon_0\boldsymbol{\chi}^{(3)}(\mathbf{r})\,\vdots\,\mathbf{E}_b\mathbf{E}_b\mathbf{E}_b. \tag{S24}$$

Using Eqs. (S10), this nonlinear polarization can be expanded as:

$$\begin{aligned}\mathbf{P}_{nl}(\omega_i) =& \frac{3\varepsilon_0}{4P_i\sqrt{P_i}}\boldsymbol{\chi}^{(3)}\vdots\mathbf{e}_i\mathbf{e}_i^*\mathbf{e}_i|A_i|^2A_ie^{i\beta_i z} + \frac{3\varepsilon_0}{2\sqrt{P_i}}\sum_{\substack{n=1\\ n\neq i}}^{4}\frac{1}{P_n}\boldsymbol{\chi}^{(3)}\vdots\mathbf{e}_n\mathbf{e}_n^*\mathbf{e}_i|A_n|^2A_ie^{i\beta_i z} \\ &+ \frac{3\varepsilon_0}{2}\sum_{n,p,q}\frac{e^{i(\beta_n-\beta_p+\beta_q)z}}{\sqrt{P_nP_pP_q}}\boldsymbol{\chi}^{(3)}\vdots\mathbf{e}_n\mathbf{e}_p^*\mathbf{e}_qA_nA_p^*A_q\Bigg|_{\substack{i\neq n\neq p\neq q\\ \omega_n-\omega_p+\omega_q=\omega_i}}, \quad i=1,2,3,4. \end{aligned} \tag{S25}$$

Inserting Eqs. (S23) and (S25) in Eq. (S21) and neglecting all rapidly varying terms (terms that are not phase matched) one obtains the following system of coupled equations describing the FWM process:

$$i\frac{dA_m}{dz} + \kappa_m(z)A_m + \gamma_m(z)|A_m|^2 A_m + \sum_{\substack{n=1\\ n\neq m}}^{4} 2\gamma_{mn}(z)|A_n|^2 A_m + \sum_{j,k,l} 2\gamma_{mnpq}(z)e^{i(\beta_n-\beta_p+\beta_q-\beta_m)z}A_n A_p^* A_q\Bigg|_{\substack{m\neq n\neq p\neq q\\ \omega_n-\omega_p+\omega_q=\omega_m}} = 0, \quad m=1,2,3,4, \tag{S26}$$

where

$$\kappa_m(z) = \frac{\epsilon_0\omega_m}{4P_m}\int_{S_\infty}\mathbf{e}_m^*\cdot\boldsymbol{\epsilon}_{NH}\mathbf{e}_m dS, \tag{S27a}$$

$$\gamma_m(z) = \frac{3\epsilon_0\omega_m}{16P_m^2}\int_{S_\infty}\mathbf{e}_m^*\cdot\boldsymbol{\chi}^{(3)}\vdots\mathbf{e}_m\mathbf{e}_m^*\mathbf{e}_m dS, \tag{S27b}$$

$$\gamma_{mn}(z) = \frac{3\epsilon_0\omega_m}{16P_mP_n}\int_{S_\infty}\mathbf{e}_m^*\cdot\boldsymbol{\chi}^{(3)}\vdots\mathbf{e}_n\mathbf{e}_n^*\mathbf{e}_m dS, \tag{S27c}$$

$$\gamma_{mnpq}(z) = \frac{3\varepsilon_0\omega_m}{16\sqrt{P_mP_nP_pP_q}}\int_{S_\infty}\mathbf{e}_m^*\cdot\boldsymbol{\chi}^{(3)}\vdots\mathbf{e}_n\mathbf{e}_p^*\mathbf{e}_q dS. \tag{S27d}$$

Note that for phase-matched or nearly phase-matched FWM interactions the factor $e^{i(\beta_n-\beta_p+\beta_q-\beta_m)z} = e^{i\Delta\beta z}$, varies only slowly with the propagation distance z as in these cases the wave vector mismatch $\Delta\beta \ll \beta_i$, $i = 1,2,3,4$. Moreover, the terms appearing in Eq. (S26) have the following physical meaning: the second term describes the propagation losses, the real and imaginary parts of the third term represent the self-phase modulation and two-photon absorption, respectively, the real and imaginary parts of the fourth term describe the cross-phase modulation and cross two-photon absorption, respectively, and the last term governs the FWM interaction.

In the case of degenerate FWM interaction, namely when $\omega_1 = \omega_2 \equiv \omega_p$, $\omega_3 \equiv \omega_s$, and $\omega_4 \equiv \omega_i$, where ω_p, ω_s, and ω_i are the frequencies of the pump, signal, and idler, respectively, the CMEs can be simplified as:

$$i\frac{dA_p}{dz} + \kappa_p(z)A_p + \left[\gamma_p(z)|A_p|^2 + 2\gamma_{ps}(z)|A_s|^2 + 2\gamma_{pi}(z)|A_i|^2\right]A_p + 2\gamma_{psi}(z)e^{i\Delta\beta z}A_sA_p^*A_i = 0, \tag{S28a}$$

$$i\frac{dA_s}{dz} + \kappa_s(z)A_s + \left[\gamma_s(z)|A_s|^2 + 2\gamma_{sp}(z)|A_p|^2 + 2\gamma_{si}(z)|A_i|^2\right]A_s + \gamma_{spi}(z)e^{-i\Delta\beta z}A_p^2A_i^* = 0, \tag{S28b}$$

$$i\frac{dA_i}{dz} + \kappa_i(z)A_i + \left[\gamma_i(z)|A_i|^2 + 2\gamma_{ip}(z)|A_p|^2 + 2\gamma_{is}(z)|A_s|^2\right]A_i + \gamma_{ips}(z)e^{-i\Delta\beta z}A_p^2A_s^* = 0, \tag{S28c}$$

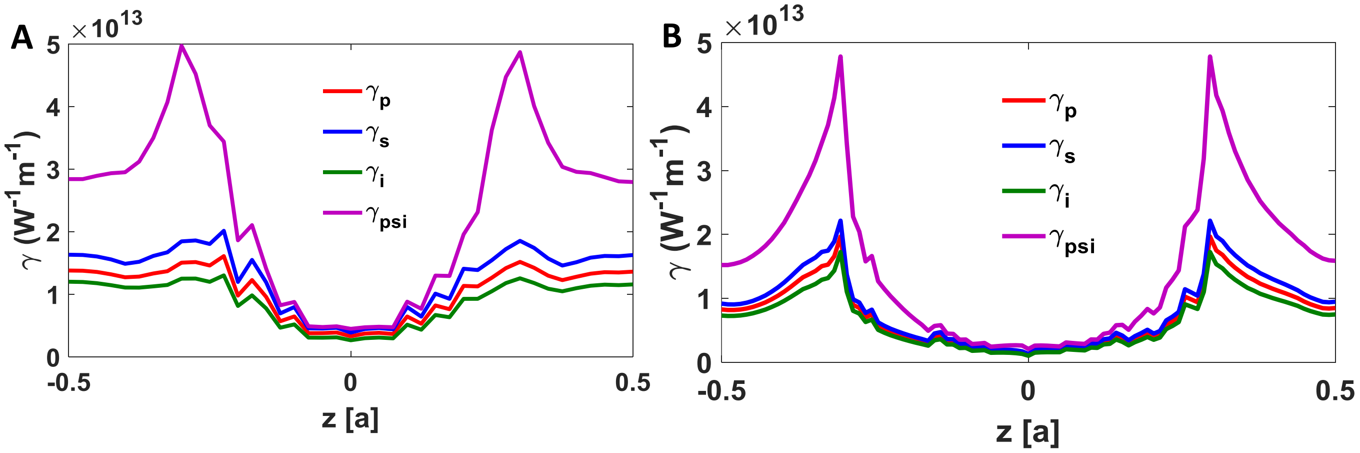

FIG. S1: **Dependence of the effective waveguide nonlinear coefficient** γ **on** z**.** (**A**) Topologically-protected graphene plasmonic mode corresponds to a freestanding metasurface. (**B**) Topologically-protected graphene plasmonic mode corresponds to a metasurface placed on a polymethyl methacrylate substrate.

where $\Delta\beta = \beta_s + \beta_i - 2\beta_p$. The coefficients of the linear and nonlinear terms are defined as:

$$\kappa_m(z) = \frac{\epsilon_0\omega_m}{4P_m}\int_{S_\infty} \mathbf{e}_m^* \cdot \epsilon_{NH}\mathbf{e}_m dS, \tag{S29a}$$

$$\gamma_m(z) = \frac{3\epsilon_0\omega_m}{16P_m^2}\int_{S_\infty} \mathbf{e}_m^* \cdot \boldsymbol{\chi}^{(3)}(\omega_m;\omega_m,-\omega_m,\omega_m)\vdots\mathbf{e}_m\mathbf{e}_m^*\mathbf{e}_m dS, \tag{S29b}$$

$$\gamma_{mn}(z) = \frac{3\epsilon_0\omega_m}{16P_mP_n}\int_{S_\infty} \mathbf{e}_m^* \cdot \boldsymbol{\chi}^{(3)}(\omega_m;\omega_n,-\omega_n,\omega_m)\vdots\mathbf{e}_n\mathbf{e}_n^*\mathbf{e}_m dS, \tag{S29c}$$

$$\gamma_{psi}(z) = \frac{3\epsilon_0\omega_p}{16P_p\sqrt{P_sP_i}}\int_{S_\infty} \mathbf{e}_p^* \cdot \boldsymbol{\chi}^{(3)}(\omega_p;\omega_s,-\omega_p,\omega_i)\vdots\mathbf{e}_s\mathbf{e}_p^*\mathbf{e}_i dS, \tag{S29d}$$

$$\gamma_{spi}(z) = \frac{3\epsilon_0\omega_s}{16P_p\sqrt{P_sP_i}}\int_{S_\infty} \mathbf{e}_s^* \cdot \boldsymbol{\chi}^{(3)}(\omega_s;\omega_p,-\omega_i,\omega_p)\vdots\mathbf{e}_p\mathbf{e}_i^*\mathbf{e}_p dS, \tag{S29e}$$

$$\gamma_{ips}(z) = \frac{3\epsilon_0\omega_i}{16P_p\sqrt{P_sP_i}}\int_{S_\infty} \mathbf{e}_i^* \cdot \boldsymbol{\chi}^{(3)}(\omega_i;\omega_p,-\omega_s,\omega_p)\vdots\mathbf{e}_p\mathbf{e}_s^*\mathbf{e}_p dS, \tag{S29f}$$

where m and $n \neq m$ indicate one of the indices p, s, and i, and the frequency degeneracy at the pump frequency has been taken into account. These modal coefficients are periodic functions with period equal to the lattice constat, a. The z-dependance of γ_m, $m = p, s, i$, and $\gamma_{psi} \equiv \gamma_{FWM}$ are presented in Fig. S1. Moreover, the characteristic length over which the field amplitudes vary is equal to $1/\Delta\beta$. When the FWM process is nearly phase-matched, we have $a \ll 1/\Delta\beta$. In this case, it is more convenient to introduce the following averaged physical quantities:

$$\bar\kappa_m = \frac{1}{a}\int_{z_0}^{z_0+a}\kappa_m(z)dz, \qquad \bar\gamma_m = \frac{1}{a}\int_{z_0}^{z_0+a}\gamma_m(z)dz, \qquad \bar\gamma_{mn} = \frac{1}{a}\int_{z_0}^{z_0+a}\gamma_{mn}(z)dz, \tag{S30a}$$

$$\bar\gamma_{psi} = \frac{1}{a}\int_{z_0}^{z_0+a}\gamma_{psi}(z)dz, \qquad \bar\gamma_{spi} = \frac{1}{a}\int_{z_0}^{z_0+a}\gamma_{spi}(z)dz, \qquad \bar\gamma_{ips} = \frac{1}{a}\int_{z_0}^{z_0+a}\kappa_m(z)dz, \tag{S30b}$$

$$\bar A_p(z) = \frac{1}{a}\int_{z-a/2}^{z+a/2}A_p(z')dz', \qquad \bar A_s(z) = \frac{1}{a}\int_{z-a/2}^{z+a/2}A_s(z')dz', \qquad \bar A_i(z) = \frac{1}{a}\int_{z-a/2}^{z+a/2}A_i(z')dz', \tag{S30c}$$

where z_0 is an arbitrary position in one unit cell. Then, by averaging Eqs. (S28), we arrive at the system of coupled-mode

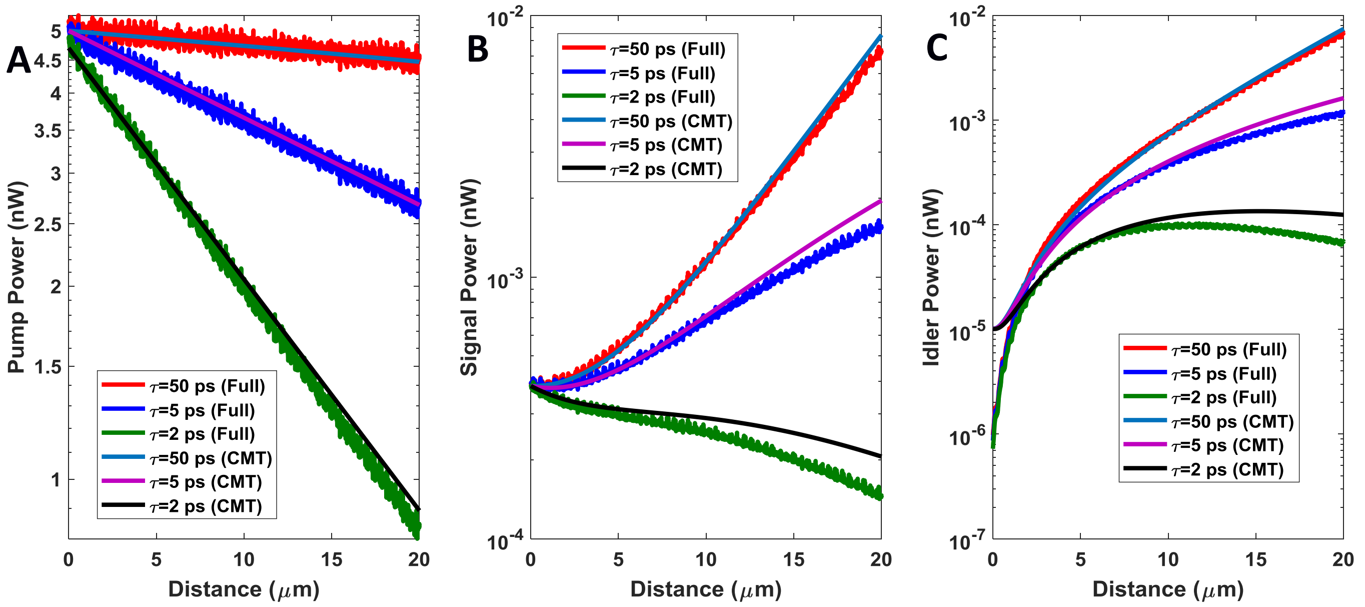

FIG. S2: **Effect of optical losses as described by the coupled-mode theory.** (**A** to **C**) Dependence of pump, signal and idler power, respectively, on the propagation distance along the system edge, determined for different values of the plasmon life-time using the coupled-mode theory and full-wave numerical simulations.

equations governing the nonlinear dynamics of the interacting modes:

$$i\frac{d\bar{A}_p}{dz}+\bar{\kappa}_p\bar{A}_p+\left(\bar{\gamma}_p|\bar{A}_p|^2+2\bar{\gamma}_{ps}|\bar{A}_s|^2+2\bar{\gamma}_{pi}|\bar{A}_i|^2\right)\bar{A}_p+2\bar{\gamma}_{psi}e^{i\Delta\beta z}\bar{A}_s\bar{A}_p^*\bar{A}_i=0, \tag{S31a}$$

$$i\frac{d\bar{A}_s}{dz}+\bar{\kappa}_s\bar{A}_s+\left(\bar{\gamma}_s|\bar{A}_s|^2+2\bar{\gamma}_{sp}|\bar{A}_p|^2+2\bar{\gamma}_{si}|\bar{A}_i|^2\right)\bar{A}_s+\bar{\gamma}_{spi}e^{-i\Delta\beta z}\bar{A}_p^2\bar{A}_i^*=0, \tag{S31b}$$

$$i\frac{d\bar{A}_i}{dz}+\bar{\kappa}_i\bar{A}_i+\left(\bar{\gamma}_i|\bar{A}_i|^2+2\bar{\gamma}_{ip}|\bar{A}_p|^2+2\bar{\gamma}_{is}|\bar{A}_s|^2\right)\bar{A}_i+\bar{\gamma}_{ips}e^{-i\Delta\beta z}\bar{A}_p^2\bar{A}_s^*=0. \tag{S31c}$$

where $\bar{A}_p$, $\bar{A}_s$, and $\bar{A}_i$ are the averaged mode amplitudes at the pump, signal, and idler frequencies, respectively.

Using full-wave numerical calculations and Eq. (S30)a and Eq. (S30)b, the nonlinearity coefficients in our examples corresponding to a freestanding graphene metasurface are $\bar{\gamma}_p = 1.1\times10^{13}\,\mathrm{W^{-1}m^{-1}}$, $\bar{\gamma}_s = 1.3\times10^{13}\,\mathrm{W^{-1}m^{-1}}$, $\bar{\gamma}_i = 0.9\times10^{13}\,\mathrm{W^{-1}m^{-1}}$, and $\bar{\gamma}_{psi}=\bar{\gamma}_{spi}=\bar{\gamma}_{ips}=\bar{\gamma}_{FWM}=2.4\times10^{13}\,\mathrm{W^{-1}m^{-1}}$.

Section S3. Influence of optical losses on graphene topological plasmonic systems

In order to verify the accuracy of the CMEs, in which losses and nonlinearities are incorporated perturbatively, we have compared the results obtained using full-wave numerical simulations and by solving the CMEs. Using Eq. (S30)a, we determined

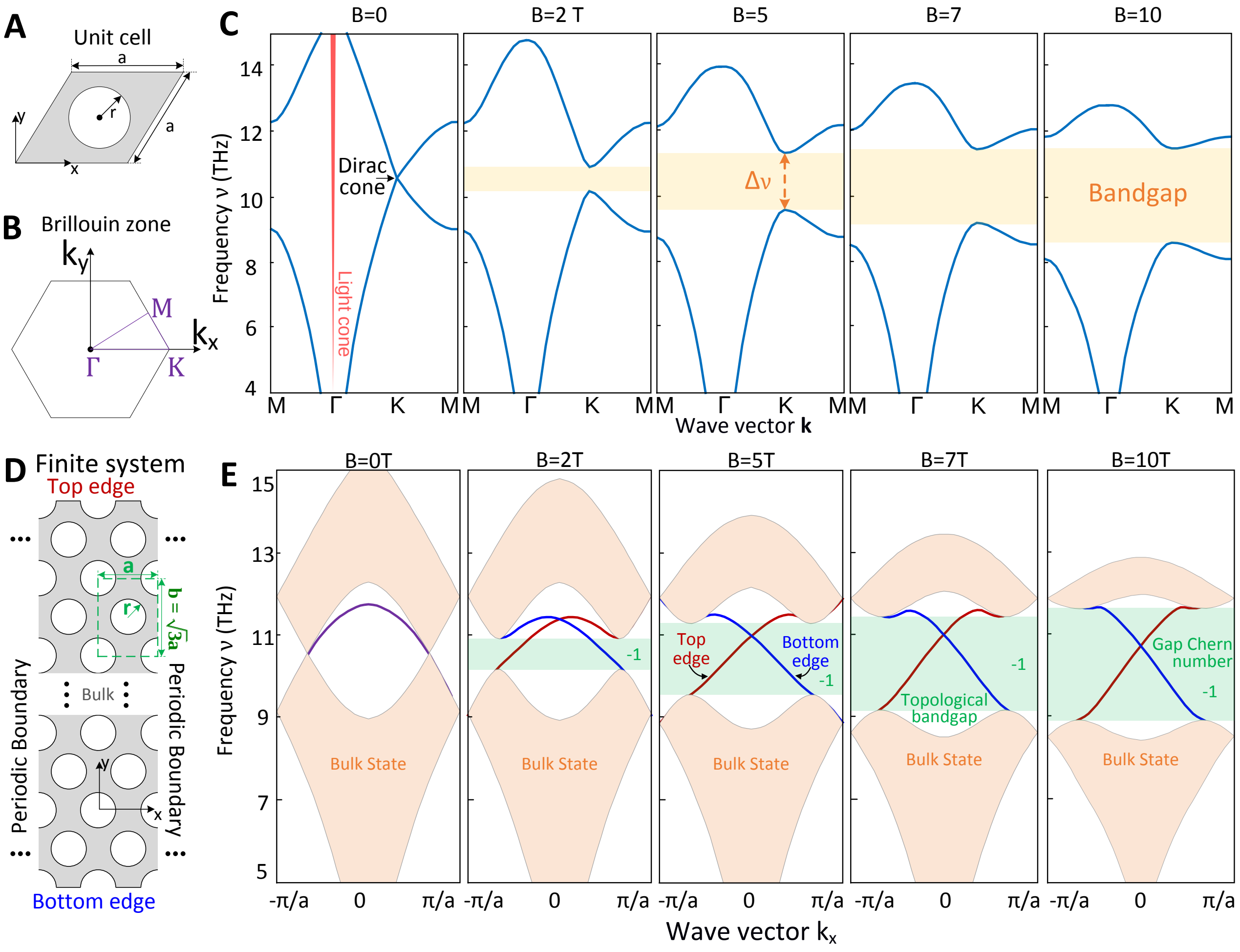

FIG. S3: **Band diagrams of a graphene metasurface on a PMMA substrate.** (**A**) Unit cell and (**B**) the first Brillouin zone. (**C**) Band diagrams of the graphene metasurface placed onto a PMMA substrate. (**D**) Geometry of a finite graphene metasurface which is finite along the y-axis and infinite along the x-axis. (**E**) Projected band diagrams of the finite metasurface placed onto a PMMA substrate and whose topological edge bands of the top and bottom edges are depicted by red and blue curves, respectively.

the loss coefficient for several values of the plasmon lifetime: $\bar{\kappa} = 2.7 \times 10^3\,\mathrm{m}^{-1}$ at $\tau = 50\,\mathrm{ps}$, $\bar{\kappa} = 15.6 \times 10^3\,\mathrm{m}^{-1}$ at $\tau = 5\,\mathrm{ps}$, and $\bar{\kappa} = 41.5 \times 10^3\,\mathrm{m}^{-1}$ at $\tau = 2\,\mathrm{ps}$.

The comparison between the results of full-wave numerical simulations and the predictions based on CMT are summarized in Fig. S2. These results reveal two important ideas: First, our perturbative approach agrees very well with full-wave simulations when the intrinsic loss of graphene is relatively weak, that is when the life-time $\tau \geq 5\,\mathrm{ps}$. Secondly, as we expect, the CMT is less accurate when the lifetime of graphene plasmons decreases, meaning that in this case optical losses can not be simply treated as a perturbation.

Section S4. Influence of the substrate on graphene topological plasmonic systems

In order to consider the effect of the substrate on the FWM interaction, we assumed that the graphene metasurface is placed onto a polymethyl methacrylate (PMMA) substrate, and computed the corresponding band diagrams. The results of these calculations are summarized in Fig. S3. They show that the main qualitative features of the topological bandgaps and the topological edge states are preserved in the presence of the substrate. The only changes are of quantitative nature, namely the frequency range of topological bandgaps shifts to lower frequency, which is a well-known phenomenon. Moreover, the effective nonlinear mode coefficients in the presence of the PMMA substrate are $\bar{\gamma}_p = 0.71 \times 10^{13}\,\mathrm{W}^{-1}\mathrm{m}^{-1}$, $\bar{\gamma}_s = 0.78 \times 10^{13}\,\mathrm{W}^{-1}\mathrm{m}^{-1}$, $\bar{\gamma}_i = 0.62 \times 10^{13}\,\mathrm{W}^{-1}\mathrm{m}^{-1}$, and $\bar{\gamma}_{psi} = \bar{\gamma}_{spi} = \bar{\gamma}_{ips} = \bar{\gamma}_{FWM} = 1.39 \times 10^{13}\,\mathrm{W}^{-1}\mathrm{m}^{-1}$. These values suggest that the effective nonlinear mode coefficients in the presence of the PMMA substrate are of the same order of magnitude as those corresponding to the case of a freestanding graphene metasurface.
